# Supplementary figures and images for: A detailed molecular network map and model of the NLRP3 inflammasome
Source: Front Immunol. 2023 Nov 15;14:1233680. doi: 10.3389/fimmu.2023.1233680 (PMC10699087; doi:10.3389/fimmu.2023.1233680)

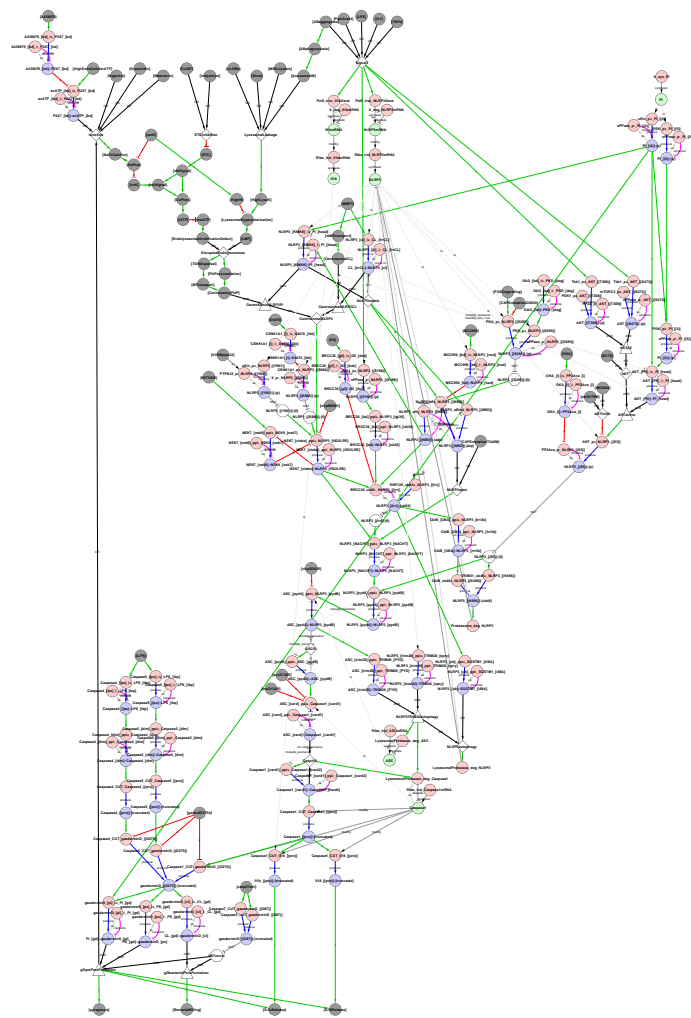

Supplement: Supplementary file 1 [file Image_1.pdf]
